# Calomel in Cardiac Dropsy

**Published:** 1890-06-14

**Authors:** 


### CALOMEL IN CARDIAC DROPSY.

Digitalis and squills, among our older drugs, and strophanthus and convallaria among later introductions, acting as direct stimulants to the muscular structure of the heart, and increasing arterial tension, thereby promoting the renal secretion, have proved so generally successful in the treatment of cardiac dropsy that the old fashioned remedy calomel has fallen into almost complete disuse. Doubtless it has been grossly abused in past time, and its neglect at present is due partly to painful recollections of salivation, &c., to a fear of inducing stomatitis, and to the want of so clear an apprehension of its physiological action as we have of that of the drugs we have named. But there seem to be cases in which it gives results that they fail to achieve, and Dr. Garvens has reported three such which leave no doubt as to the error of neglecting so powerful an agent altogether. They all occurred within the last two years in his wards in the Masonic Hospital at Hamburg. The first a man, aged 43, suffered from myocarditis, arterial sclerosis, enormous ascites, intense œdema of the lower extremities, and some anasarca of the upper part of the body, with effusion into the right pleura. The heart was much displaced and its sounds weak, the pulse feeble, irregular, and unequal. The urine was albumenous though without casts. There was great dyspnœa, and the patient's mind was confused. For a week Dr. Garvens tried digitalis, strophanthus and sodio-salicylate of caffein without any benefit. He then gave calomel in doses of gr. iii, four times a day, increasing it shortly afterwards to five times. The excretion of urine, which had been not more than 600 cub. centims. was somewhat less during the first three days of the new treatment, but rose on the fourth to 2,400, on the fifth to 6,000, and was on the sixth 5,600. The saliva, sweat, and tears were profuse. The calomel was then discontinued, and the urine fell to 3,200 cms. on the seventh, and gradually declined to 1,200, at which it remained constant for some time. By the tenth day the dropsy had almost entirely disappeared, the man's appetite and sleep were good, and his general state satisfactory. At the end of the month the dropsy reappeared as before, together with a like diminution of the urine to 600 cms. The calomel was resumed, the urine rose to 5,500, and the anasarca and ascites passed away. Digitalis and tonics now acted better, the excretion of urine was not under 1,000 cms. and the patient was discharged feeling quite well ten weeks from his first admission. Twice again, however, during the winter he presented himself with the old symptoms, to be relieved as rapidly by the calomel, but on the fifth occasion he succumbed.

Two other cases were even more gratifying, as in these there had been no relapse for several months. The symptoms were much the same as those described, but one patient, aged 53, was a hard drinker, had hepatic cirrhosis, though no hydrothorax or albumenuria. He had a large ulcer on his right leg, and when admitted was apparently dying. As digitalis had already been given without effect he was at once put on the calomel treatment, 1½ grains every two hours. This was continued for ten days; the urine which measured 250 cms. only on the second day rose to 1,200 on the fourth, and 5,200 on the sixth, when, with moderate diarrhœa and some stomatitis, for which a gargle of chlorate of potash was given, the dropsy and ascites, as well as the dyspnœa, were fast disappearing. During the following four days of the calomel treatment the urine fell to 3,500, 3,000, 1,600, and 1,200 cms., at which it remained constant. He was now quite well, and after a further period of ten days' treatment with digitalis only he was discharged. The last case was fifty-eight years of age, and, like the first, had pleuritic effusion and albumenuria, but was almost unconscious when admitted. The orthopnœa was so extreme that tapping of the abdomen was performed to avert imminent death. Next day the calomel treatment, gr. 1½ every two hours, was commenced. The urine, then 250 cms., rose to 700, 2,800, and 4,600 cms. when on the fourth day he first awoke to consciousness. The dropsy and dyspnœa were now rapidly improving, and by the eighth day had entirely gone. The calomel was then discontinued, the urine fell gradually to 1,600 cms., and the patient felt remarkably well, with an excellent appetite. He left at the end of five weeks, but returned some time later to have the fluid removed from the the pleura by aspiration, since it interfered somewhat with his respiration.

Dr. Garvens observes that in none of these cases was the diarrhœa excessive, or the stomatitis severe enough to cause any complaints on the part of the patients, and calls attention to the somewhat remarkable fact that while in all the anasarca and ascites yielded to the calomel with surprising rapidity, the pleuritic effusion, present in the first and third, was uninfluenced by it. This is the more difficult of explanation, as he is inclined to agree with those who maintain that its action is not so much on the kidneys as on the lymphatic system in general. That it is not simply a diuretic is shown by the falling off in the renal excretion after a few days of enormous increase while the drug is still being administered.

Anyhow, calomel in these cases is one of those "good remedies—out of fashion," of which Dr. Hare wrote, and these three cases are sufficient to prove its value under circumstances of an apparently hopeless character.

